# Surveillance intensity influences the observed incidence of delayed hyponatremia after microscopic transsphenoidal pituitary surgery: early sodium and sex as risk stratifiers

**DOI:** 10.3389/fendo.2026.1946832

**Published:** 2026-09-24

**Authors:** Müjdat Kara, Ece Cıllı, Serhat Özfıstıkçı, Yiğit Nazım Kara, İlham Gafarlı, Ömer Orhun

**Affiliations:** 1 Department of Endocrinology, School of Medicine, Acibadem Mehmet Ali Aydinlar University, Istanbul, Türkiye; 2 School of Medicine, Acibadem Mehmet Ali Aydinlar University, Istanbul, Türkiye; 3 School of Medicine, Dokuz Eylül University, İzmir, Türkiye; 4 Kanuni Sultan Süleyman Training and Research Hospital, University of Health Sciences, Istanbul, Türkiye; 5 Department of Neurosurgery, Cerrahpaşa School of Medicine, Istanbul University-Cerrahpaşa, Istanbul, Türkiye

**Keywords:** ascertainment bias, bedside predictor, delayed postoperative hyponatremia, early postoperative sodium, pituitary adenoma, sodium surveillance, transsphenoidal surgery

## Abstract

Delayed postoperative hyponatremia after transsphenoidal pituitary surgery has a reported incidence of 5–30%, yet how much of this variability reflects biology rather than differences in postoperative sodium surveillance has not been quantified within a single cohort. We retrospectively studied 285 consecutive adults who underwent microscopic transsphenoidal surgery for pituitary adenoma by a single team between 2004 and 2025. Delayed hyponatremia was defined from raw laboratory data as any serum sodium below 135 mmol/L on postoperative days 3–10. Two predefined populations were analyzed: the primary cohort (n = 281) and a surveillance-complete subgroup with at least one measurement on days 5–7 (n = 107). Apparent incidence rose from 16.4% in the primary cohort to 35.5% in the surveillance-complete subgroup and 57.5% with a day-7 measurement; those tested on days 5–7 had 11.4-fold higher odds of recorded delayed hyponatremia than those without late testing (p < 0.001). An apparent two-decade increase largely disappeared once surveillance was held constant: under the assumptions of a direct-standardization analysis, approximately 83% of the observed temporal increase was explained by differences in surveillance intensity. Early postoperative hyponatremia (any sodium below 135 mmol/L on days 0–2) showed the largest univariable association among bedside-available variables: 46.9% of affected patients later developed delayed hyponatremia versus 12.4% without it (relative risk 3.77; odds ratio 6.14), although this did not reach significance after multivariable adjustment within the surveillance-complete subgroup (odds ratio 2.83; 95% CI 0.86–9.33). Female sex was independently associated with the outcome (adjusted odds ratio 2.73), and a significant age-by-sex interaction (p = 0.010) showed consistently elevated risk in women of all ages and risk rising with age in men; age was analyzed continuously. A secondary, exploratory risk-stratification model reached an optimism-corrected area under the curve of 0.645, adequate for hypothesis generation but not clinical deployment. Even within a single surgical team, apparent incidence varied more than threefold with surveillance intensity, suggesting that much of the reported heterogeneity may reflect differences in how the outcome is ascertained rather than between patients. Whether early sodium, sex, and age can direct day 5–7 surveillance instead of screening every patient requires prospective evaluation.

## 1 Introduction

Delayed postoperative hyponatremia is the most common metabolic complication after transsphenoidal pituitary surgery and remains one of the leading causes of unplanned hospital readmission after surgery (1). Reported incidence rates vary widely, ranging from approximately 5% to 30%, and this five- to sixfold variation has remained unexplained despite more than two decades of published studies (2–6). Differences in surgical approach, patient selection, biochemical definitions, post-discharge sodium monitoring, glucocorticoid replacement, and diabetes insipidus management have all been proposed as potential explanations (7–11). However, the relative contribution of these factors has not been formally evaluated within a single surgical cohort.

From a clinical perspective, two issues deserve particular attention. First, postoperative sodium surveillance beyond postoperative day (POD) 2 is highly variable across institutions. In many centers, routine inpatient sodium measurements are limited to the first 48–72 hours after surgery, whereas outpatient sodium testing around POD7 is performed inconsistently. Because serum sodium typically reaches its lowest level between POD5 and POD8 after pituitary surgery, cohorts without systematic late sodium measurements will inevitably underestimate the true incidence of delayed postoperative hyponatremia. Despite this, the effect of surveillance intensity on the apparent incidence of delayed hyponatremia has never been quantified directly.

Second, although several prediction models have been proposed, most rely either on variables that become available relatively late during hospitalization, such as POD3–4 sodium concentrations, or on variables that overlap with the developing outcome itself, such as a POD4 sodium level below 135 mmol/L. A predictor available within the first 48 postoperative hours, before most patients are discharged, would be considerably more useful for identifying patients who require targeted outpatient sodium monitoring during POD5–7.

In this 21-year single-team cohort of microscopic transsphenoidal surgery, we addressed one primary and two secondary questions. The primary question was quantitative and concerned ascertainment: when surgical technique and the operating team remain constant, to what extent does the intensity of postoperative sodium surveillance influence the observed incidence of delayed postoperative hyponatremia? The secondary questions concerned risk stratification and are exploratory: first, can early postoperative hyponatremia, defined as any serum sodium concentration below 135 mmol/L during POD0–2, serve as a practical bedside marker of subsequent delayed hyponatremia; and second, can a simple model based on sex, baseline serum sodium, and early postoperative hyponatremia provide useful risk discrimination after internal validation? We emphasize this ordering because the two components are not of equal evidentiary weight. The surveillance analysis is the substantive contribution of this study and rests on the full cohort; the risk-stratification analysis is developed in a smaller, clinician-selected subgroup, achieves only modest discrimination, and is presented as hypothesis-generating rather than as a tool ready for clinical use.

Because all procedures in this series were performed by a single surgical team using a microscopic transsphenoidal technique, comparisons with endoscopic surgery were beyond the scope of this study. This design minimized surgical technique as a source of heterogeneity when evaluating the impact of sodium surveillance, although it may limit the generalizability of our findings to centers using mixed surgical approaches.

## 2 Materials and methods

### 2.1 Study design and study population

This retrospective single-center cohort study included consecutive adult patients (≥18 years) who underwent microscopic transsphenoidal surgery for histopathologically confirmed pituitary adenoma at a tertiary referral center between January 1, 2004, and March 31, 2025. All procedures were performed by the same dedicated pituitary surgery team, which exclusively used a microscopic transsphenoidal technique throughout the study period. No endoscopic procedures were included.

Patients with non-adenomatous sellar lesions, including Rathke’s cleft cysts, craniopharyngiomas, meningiomas, metastases, and pituicytomas, were excluded based on histopathological diagnosis. During re-verification of the source records for this revision, five patients were identified whose histopathology had been recorded under a non-functioning descriptor with the non-adenomatous subtype given parenthetically (three Rathke’s cleft cysts, one adamantinomatous craniopharyngioma, one pituicytoma) and who had consequently been retained in the analysis dataset. Rather than alter the pre-specified cohort after review, these patients were kept in the primary analysis and a sensitivity analysis excluding them is reported in Section 3.12.

The study was conducted in accordance with the Declaration of Helsinki and was approved by the Medical Research Ethics Committee of Acibadem Mehmet Ali Aydinlar University. The requirement for informed consent was waived because of the retrospective design. The study was reported in accordance with the STROBE guideline for observational studies (12). Because a multivariable risk model was developed, the reporting of that component follows the TRIPOD statement for prediction model development (13): the source of data, participants, outcome, predictors, sample size, missing-data handling, statistical analysis methods, model specification, model performance, and internal validation are reported in the corresponding sections below, and no model was externally validated. Completed STROBE and TRIPOD checklists are provided as **Supplementary Material**, and a **Supplementary Methods** appendix gives the operational variable definitions, the penalized-likelihood algorithm, and the resampling procedures in sufficient detail to permit independent replication.

### 2.2 Outcome definitions

The primary outcome was delayed postoperative hyponatremia, defined directly from raw laboratory data as any serum sodium concentration <135 mmol/L recorded between postoperative days (POD) 3 and 10. The outcome was derived from laboratory measurements rather than from a predefined database variable.

During data verification, the original database variable for delayed hyponatremia was found to be inconsistent with the laboratory-derived definition in 20 patients. Therefore, the laboratory-based definition was adopted as the primary outcome throughout the study.

Secondary outcomes included:

- severe delayed postoperative hyponatremia (serum sodium <130 mmol/L during POD3–10);
- profound delayed postoperative hyponatremia (serum sodium <125 mmol/L during POD3–10); and
- postoperative sodium trajectory, classified as no hyponatremia, early-only (POD0–2 sodium <135 mmol/L without delayed hyponatremia), delayed-only (delayed hyponatremia with normal POD0–2 sodium), or biphasic (both early and delayed hyponatremia).

Because serum and urine osmolality, urine sodium, volume status, and adrenal and thyroid function tests were not consistently available throughout the 21-year study period, outcomes were not adjudicated as the syndrome of inappropriate antidiuretic hormone secretion (SIADH). Accordingly, the more conservative term delayed postoperative hyponatremia is used throughout this manuscript. In neurosurgical populations in which such adjudication has been carried out, SIADH accounts for the majority of severe hyponatremia (8), but we did not have the data required to assign a mechanism in individual patients and have therefore made no claim about the pathophysiology underlying the events reported here.

### 2.3 Analysis populations

Given the substantial variation in postoperative sodium surveillance identified during the initial data review, two analysis populations were predefined.

The primary analysis cohort included all patients with at least one serum sodium measurement between POD3 and POD10 (n = 281). This represented the largest analyzable cohort but included heterogeneous postoperative surveillance.

The surveillance-complete subgroup included patients with at least one serum sodium measurement between POD5 and POD7 (n = 107). Because this interval encompasses the expected biological nadir of postoperative sodium, this subgroup was considered the population with the most complete outcome ascertainment and the lowest risk of outcome misclassification, and it served as the development cohort for multivariable prediction modeling. This subgroup is not, however, an unbiased sample of the overall postoperative population. Receipt of POD5–7 testing was determined by clinical practice rather than by protocol, so these patients were more intensively monitored and may differ systematically from patients discharged before POD5. Models developed in this subgroup are therefore reported as internally valid within a completely ascertained population rather than as directly generalizable to all operated patients.

Both populations were analyzed in parallel. Differences between them were interpreted as quantitative estimates of surveillance-related ascertainment bias.

### 2.4 Candidate predictors

Candidate predictors were selected *a priori* based on clinical availability before hospital discharge and previous literature.

The predefined candidate variables included age (continuous), female sex, preoperative serum sodium concentration, the change in serum sodium from the preoperative value to POD2, early postoperative hyponatremia (any serum sodium <135 mmol/L during POD0–2), macroadenoma status, optic chiasm compression, cavernous sinus invasion, supra- or extrasellar extension, previous pituitary surgery, previous Gamma Knife radiosurgery or radiotherapy, and histopathological cell lineage.

A proxy variable for functional adenoma was also evaluated. Corticotroph, somatotroph, lactotroph, and plurihormonal/mixed adenomas were classified as functional, whereas gonadotroph and non-functioning adenomas were classified as non-functioning.

Length of hospital stay was intentionally excluded as a predictor because it lies on the causal pathway between exposure and outcome and is influenced by outcome detection, introducing both reverse causation and informative observation bias. Instead, it was analyzed only as a descriptive secondary outcome.

The year of surgery was examined both as a continuous variable (per 5-year increase) and as predefined calendar periods (2004–2010, 2011–2017, and 2018–2025). Because exploratory analyses demonstrated substantial confounding by surveillance intensity, calendar period was retained only for descriptive analyses and was not included in the primary prediction model.

### 2.5 Handling of missing data

Patients with no POD3–10 sodium measurement (n=4) were excluded from the primary outcome analysis; the outcome was not imputed. For continuous predictors with <10% missingness, complete-case analysis was used. Missingness of late sodium measurements (POD5–10) was treated as informative and was itself analyzed as an exposure (Section 2.6, Model C); it was not imputed under any missing-at-random assumption.

One date entry containing the obvious typographical year ‘1010’ was corrected to 2010 by reference to its sequential record number and adjacent surgical dates.

### 2.6 Statistical analysis

Continuous variables were summarized as mean ± SD when approximately symmetric or median (interquartile range) otherwise; categorical variables were summarized as counts and percentages. Group comparisons used the Mann–Whitney U test, Student t-test, χ² test, or Fisher exact test as appropriate.

Univariable associations with delayed hyponatremia were estimated using Firth penalized logistic regression (14) to mitigate sparse-data bias and complete or quasi-complete separation. Multivariable models were fit with the same penalized approach. The number of covariates in each model was fixed *a priori* so that at least eight events per variable were retained; the resulting events-per-variable (EPV) values were 9.0 for Model A (36 events, 4 covariates), 12.0 for Model B (36 events, 3 covariates), and 11.0 for Model C (44 events, 4 covariates). Because Model A falls below the conventional EPV threshold of 10 recommended for unpenalized maximum-likelihood logistic regression, and because several candidate covariates were sparse, Firth penalization was applied to all models rather than being reserved for models exhibiting separation. This choice was made in advance on the basis of the design of the study and not after inspecting model output. Three multivariable models were specified in advance and are all reported in full. They were not treated as competing candidates from which a single best-performing model was to be selected; each addresses a different question, and no model was chosen, dropped, or reordered on the basis of its discrimination, calibration, decision-curve performance, or the statistical significance of its coefficients:

- Model A (primary, sentinel model): female sex, preoperative sodium, early hyponatremia (POD0–2), functional-adenoma proxy. Fitted on the surveillance-complete subgroup.
- Model B (sodium-trajectory variant): female sex, preoperative-to-POD2 sodium decline, early hyponatremia. Fitted on the surveillance-complete subgroup.
- Model C (sensitivity, full-cohort): female sex, preoperative sodium, early hyponatremia, and a binary surveillance flag (POD5–7 measurement available) to demonstrate the magnitude of surveillance-driven detection. Model A was designated the primary model before analysis because the outcome can only be observed in patients in whom sodium was actually measured during the interval containing its biological nadir. In the primary cohort, patients without a POD5–7 measurement are recorded as event-free whether or not delayed hyponatremia occurred, so a model developed in that population would be fitted against an outcome that is systematically misclassified in a large fraction of patients, and its coefficients would reflect the determinants of being tested as much as the determinants of the outcome. Restricting model development to the surveillance-complete subgroup removes that misclassification at the cost of generalizability, and Model C is reported alongside it precisely so that the trade-off is visible: it retains every patient and carries the surveillance indicator as an explicit covariate. The two models are therefore complementary rather than alternatives, and both are reported irrespective of their performance.

### 2.7 Model performance and internal validation

Discrimination was assessed by the area under the receiver operating characteristic curve (AUC) with 2,000-resample percentile bootstrap 95% confidence intervals. Calibration was assessed by intercept, slope, and graphical inspection; the Brier score was compared with the null Brier score ( $\bar{\pi }$·(1− $\bar{\pi }$), where $\bar{\pi }$ is the marginal event rate) to quantify absolute improvement beyond predicting the baseline incidence. Internal validation used the same number of bootstrap resamples (2,000) to estimate optimism in AUC, Brier score, and calibration slope; corrected metrics were reported as apparent − optimism. The percentile confidence interval and the optimism correction are two distinct procedures rather than two runs of one procedure: the former resamples the dataset to quantify the sampling variability of the apparent estimate, whereas the latter refits the model within every resample and applies it back to the original data to quantify overfitting. Both are now based on 2,000 resamples so that a single resampling scheme underlies all reported performance metrics. Clinical utility was assessed using decision-curve analysis (DCA) across threshold probabilities of 5–70%.

All analyses were performed in Python 3.12 (pandas 2.2, scipy 1.17, scikit-learn 1.6, NumPy 2.3) with Firth penalized logistic regression implemented by Newton–Raphson optimization on the modified score equation (verified against published worked examples). Two-sided p-values <0.05 were considered statistically significant; given the exploratory nature of secondary and subgroup analyses, no formal multiple-testing correction was applied and these p-values are interpreted as descriptive.

## 3 Results

### 3.1 Cohort flow and baseline characteristics

Among 285 consecutive patients, four had no serum sodium measurements between POD3 and POD10 and were therefore excluded from the primary outcome analysis. The final primary cohort consisted of 281 patients. Of these, 107 had at least one sodium measurement during POD5–7 and comprised the surveillance-complete subgroup. Participant flow, including the analysis populations and the complete-case samples used for each multivariable model, is shown in **Figure 1**.

**Figure 1:**
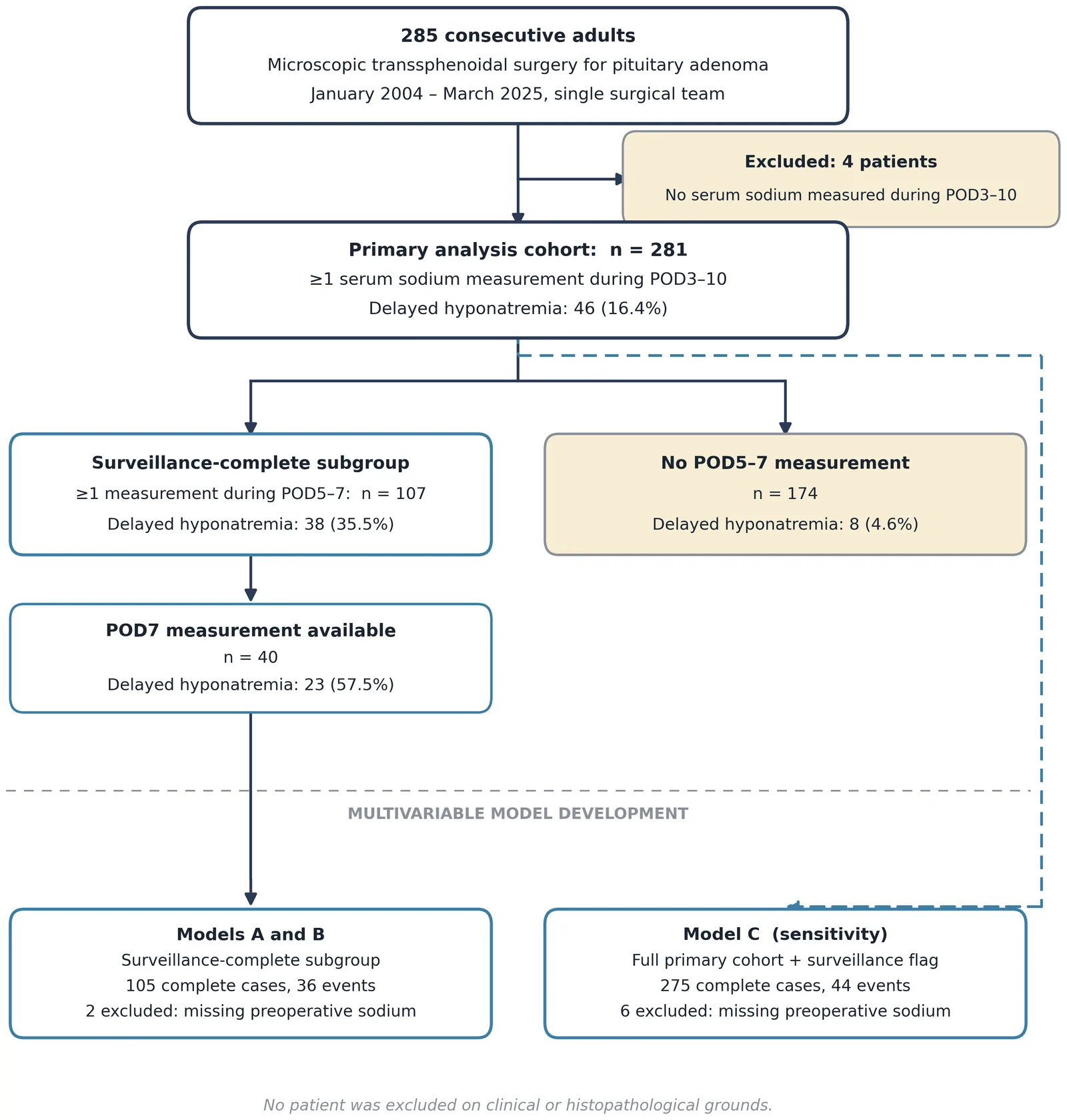
Participant flow. Of 285 consecutive adults who underwent microscopic transsphenoidal surgery for histopathologically confirmed pituitary adenoma between January 2004 and March 2025, four had no serum sodium measurement between POD3 and POD10 and could not contribute to the primary outcome. The primary analysis cohort therefore comprised 281 patients, among whom 46 (16.4%) developed delayed postoperative hyponatremia. Within this cohort, 107 patients had at least one sodium measurement during POD5–7 and formed the surveillance-complete subgroup (38 events; 35.5%), whereas 174 patients had no POD5–7 measurement (8 events; 4.6%); of the surveillance-complete subgroup, 40 patients had a POD7 measurement (23 events; 57.5%). Models A and B were developed in the surveillance-complete subgroup using 105 complete cases with 36 events, after exclusion of two patients with missing preoperative sodium; Model C was fitted in the full primary cohort using 275 complete cases with 44 events. No patient was excluded on clinical or histopathological grounds.

Delayed postoperative hyponatremia (serum sodium <135 mmol/L during POD3–10) occurred in 46 of 281 patients (16.4%) in the primary cohort and in 38 of 107 patients (35.5%) in the surveillance-complete subgroup.

The four excluded patients were omitted solely because no sodium measurements were available during the outcome assessment period; no patients were excluded on clinical or histopathological grounds.

The mean age of the study population was 44.8 ± 14.8 years, and 122 patients (43.4%) were women. Among patients with available radiological data, 97% had macroadenomas, reflecting the referral pattern of this tertiary center. The most common histopathological subtypes were gonadotroph adenomas (27.0%), somatotroph adenomas (19.9%), and non-functioning adenomas (19.9%), whereas corticotroph adenomas accounted for 10.0% of the cohort.

Baseline characteristics according to delayed postoperative hyponatremia status are summarized in **Table 1**.

**Table 1:** Baseline characteristics and univariable associations with delayed postoperative hyponatremia in the primary cohort (n = 281).

Characteristic	No delayed hyponatremia (n = 235)	Delayed hyponatremia (n = 46)	Univariable OR (95% CI)	p
Demographics				
Age, years	44.5 ± 14.6	46.7 ± 15.5	1.11 per 10 yr (0.90–1.37)	0.35
**Female sex**	**93 (39.6%)**	**29 (63.0%)**	**2.57 (1.34–4.91)**	**0.004**
**Preoperative serum Na, mmol/L**	**139.4 (138.0–141.0)**	**138.7 (137.0–140.0)**	**0.88 per mmol/L (0.78–0.99)**	**0.033**
Preoperative hyponatremia (<135 mmol/L)	10 (4.3%)	2 (4.3%)	1.21 (0.28–5.25)	0.80
Tumor characteristics				
Macroadenoma	221/229 (96.5%)	40/40 (100.0%)	3.11 (0.15–65.23)	0.47
**Suprasellar/extrasellar extension**	**110/231 (47.6%)**	**31/45 (68.9%)**	**2.39 (1.22–4.69)**	**0.012**
**Optic chiasm compression**	**110/231 (47.6%)**	**29/45 (64.4%)**	**1.97 (1.02–3.79)**	**0.044**
Cavernous sinus invasion	106/230 (46.1%)	23/45 (51.1%)	1.22 (0.65–2.31)	0.54
Functional adenoma (proxy)	122 (51.9%)	25 (54.3%)	1.10 (0.59–2.06)	0.77
Surgical history				
Prior pituitary surgery	35 (14.9%)	7 (15.2%)	1.07 (0.45–2.55)	0.88
Prior gamma knife radiosurgery or radiotherapy	55 (23.4%)	7 (15.2%)	0.62 (0.27–1.43)	0.26
Perioperative course				
**Early hyponatremia (POD0–2 <135 mmol/L)**	**17 (7.2%)**	**15 (32.6%)**	**6.14 (2.79–13.52)**	**<0.001**

Continuous variables are mean ± SD when approximately symmetric or median (interquartile range) otherwise; categorical variables are n (%). Where radiological or histopathological data were incomplete, the denominator of non-missing observations is shown explicitly. Bold denotes p < 0.05. All odds ratios were estimated by Firth penalized univariable logistic regression with Wald 95% confidence intervals, applied uniformly to every variable in the table so that a single estimator underlies all reported effects. Penalization was used by design rather than being reserved for variables showing sparse-data behavior; the macroadenoma row illustrates its effect, since all 40 patients with radiological data in the delayed group had macroadenomas and the unpenalized estimate would be undefined. Functional adenoma proxy = corticotroph, somatotroph, lactotroph, and plurihormonal/mixed lineage; gonadotroph and non-functioning adenomas were classified as non-functioning. CI, confidence interval; OR, odds ratio; POD, postoperative day.

### 3.2 Surveillance intensity and the apparent incidence of delayed postoperative hyponatremia

The apparent incidence of delayed postoperative hyponatremia varied more than threefold across strata defined by the intensity of postoperative sodium surveillance.

Among 174 patients without a sodium measurement during POD5–7, only eight cases (4.6%) of delayed hyponatremia were identified. In contrast, 38 of 107 patients (35.5%) with at least one sodium measurement during POD5–7 developed delayed hyponatremia (Fisher’s exact OR 11.4, p<0.001).

When the analysis was restricted to the 40 patients with an available POD7 sodium measurement, the apparent incidence increased further to 57.5% (23/40).

Because the surgical team, operative technique, and patient selection remained essentially unchanged throughout the study period, this marked gradient is consistent with differences in surveillance intensity rather than with biological differences between patient groups. This study is observational and receipt of late sodium testing was determined by clinical practice rather than by design; the gradient therefore represents an association between measurement intensity and recorded incidence, and a causal contribution of unmeasured differences between tested and untested patients cannot be excluded.

The number of sodium measurements obtained during POD3–10 was the strongest correlate of recorded delayed hyponatremia. Patients without delayed hyponatremia had a median of 2 measurements (IQR, 2–3), whereas those with delayed hyponatremia had a median of 5 measurements (IQR, 4–7; Mann–Whitney p<10⁻¹⁴). Likewise, a POD7 sodium measurement was available in 50.0% of patients with delayed hyponatremia compared with only 7.2% of those without the outcome.

### 3.3 Apparent temporal trends are largely explained by differences in surveillance intensity

The crude incidence of delayed postoperative hyponatremia was 11.7% during 2004–2010, 9.6% during 2011–2017, and 27.4% during 2018–2025; the trend across the three periods was therefore not monotonic, with the rise confined to the most recent period. During the same periods, however, the proportion of patients who underwent sodium measurement during POD5–7 also increased progressively, from 22% to 39% and 55%, respectively.

When the analysis was restricted to the surveillance-complete subgroup, the apparent temporal trend became substantially less pronounced. The corresponding incidences were 34.8%, 25.0%, and 42.3% across the three periods, with no consistent temporal pattern. These findings suggest that the apparent increase in delayed postoperative hyponatremia largely reflects more intensive postoperative sodium surveillance rather than a true increase in biological event rates.

To further evaluate this observation, we performed an exploratory breakpoint analysis by iteratively testing candidate calendar years and identifying the transition associated with the greatest difference in POD5–7 surveillance. The strongest separation was observed at the 2011/2012 transition. Patients treated during 2004–2011 (Era 1; *n* = 117) had a median of two sodium measurements during POD3–10, and only 20.5% underwent sodium testing during POD5–7. In contrast, patients treated during 2012–2025 (Era 2; *n* = 164) had a median of three sodium measurements, and 50.6% underwent late sodium surveillance (Fisher’s exact OR, 3.97; 95% CI, 2.34–6.78; *p* = 2.6×10⁻⁷; Mann–Whitney *p* = 6.6×10⁻¹² for the number of measurements).

Across the same transition, the recorded incidence of delayed postoperative hyponatremia increased from 10.3% (12/117) to 20.7% (34/164) (OR, 2.23; 95% CI, 1.11–4.49; *p* = 0.022). Likewise, the overall incidence of postoperative hyponatremia (early or delayed) increased from 15.4% to 27.4% (OR, 2.05; *p* = 0.020). However, when the comparison was limited to the surveillance-complete subgroup, no significant differences were observed between the two periods. Delayed postoperative hyponatremia occurred in 33.3% and 36.1% of patients (*p* = 1.00), early postoperative hyponatremia in 20.8% and 15.7% (*p* = 0.55), and overall postoperative hyponatremia in 41.7% and 42.2% (*p* = 1.00) during Eras 1 and 2, respectively (**Table 2**).

**Table 2:** Apparent versus surveillance-controlled temporal trend in the incidence of delayed hyponatremia.

Operative era	n	POD5–7 Na measured, n (%)	Apparent incidence (whole cohort)	Incidence among measured
Era 1 (2004–2011)	117	24 (21%)	10.3% (12/117)	33.3% (8/24)
Era 2 (2012–2025)	164	83 (51%)	20.7% (34/164)	36.1% (30/83)

Surveillance-complete = patients with at least one POD5–7 sodium measurement. In the whole cohort the apparent incidence roughly doubles between eras; when monitoring is held constant (incidence among measured) the difference disappears (Fisher exact p = 1.00). Under the assumptions of direct standardization, 8.7 percentage points (≈83%) of the 10.5-point inter-era apparent rise are explained by expanded POD5–7 surveillance and 1.7 points (≈17%) are not explained by surveillance; the full decomposition and its assumptions are given in **Table 3**. Non-measurement at POD5–7 is almost entirely a consequence of discharge before POD5 (median length of stay 4 vs. 6 days); the cross-sectional measured-versus-unmeasured contrast is therefore confounded by discharge timing and is not used as the primary comparison. POD, postoperative day.

To quantify the contribution of surveillance to the apparent increase in incidence, direct standardization was performed. If Era 1 had been observed under the surveillance intensity of Era 2, the expected incidence of delayed postoperative hyponatremia would have increased from 10.3% to 19.0%. Accordingly, under the assumptions of this analysis, 8.7 percentage points (approximately 83%) of the observed 10.5-percentage-point increase were explained by differences in surveillance intensity, whereas 1.7 percentage points (approximately 17%) were not explained by surveillance. The full arithmetic of this decomposition, together with the assumptions on which it rests, is set out in **Table 3**. Direct standardization assumes that the stratum-specific incidences observed in Era 1 would have applied had those patients been monitored with the surveillance distribution of Era 2, and that measured and unmeasured patients within an era are exchangeable conditional on measurement status. Because receipt of POD5–7 testing was determined by clinical practice rather than by design, and is closely tied to discharge timing, these assumptions cannot be verified in these data. The residual 17% should therefore not be interpreted as a quantified biological effect, nor the 83% as a causal decomposition of the temporal rise into detection and biology; what the data support is that more intensive surveillance detects more delayed hyponatremia, and that the observed temporal rise is largely explained by the expansion of surveillance under a standard and explicitly stated set of assumptions.

**Table 3:** Decomposition of the apparent inter-era rise in the recorded incidence of delayed postoperative hyponatremia by direct standardization for POD5–7 sodium surveillance (primary cohort, n = 281).

Operative era	n	POD5–7 Na measured, n (%)	Incidence among measured, % (n/N)	Incidence among unmeasured, % (n/N)	Apparent incidence, whole cohort, % (n/N)
Era 1 (2004–2011)	117	24 (20.5)	33.3 (8/24)	4.3 (4/93)	10.3 (12/117)
Era 2 (2012–2025)	164	83 (50.6)	36.1 (30/83)	4.9 (4/81)	20.7 (34/164)
Era 1, standardized to the Era 2 surveillance distribution	117	— (50.6)	33.3	4.3	19.0

Standardization. The stratum-specific incidences observed in Era 1 — 33.3% among patients with at least one POD5–7 sodium measurement and 4.3% among those without — were reweighted to the surveillance distribution observed in Era 2, in which 50.6% of patients were measured. The standardized Era 1 incidence is (0.506 × 33.3) + (0.494 × 4.3) = 19.0%.

Decomposition. The apparent incidence rose by 10.5 percentage points between eras (10.3% to 20.7%). Of this, 8.7 percentage points (19.0 − 10.3; ≈83%) are accounted for by the change in the surveillance distribution alone, and 1.7 percentage points (20.7 − 19.0; ≈17%) are not accounted for by surveillance.

Supporting comparisons. Uptake of POD5–7 surveillance rose markedly between eras (20.5% to 50.6%; odds ratio 3.97; 95% CI 2.34–6.78; p = 2.6 × 10⁻⁷), whereas the incidence among measured patients did not differ (33.3% vs 36.1%; Fisher exact p = 1.00). The incidence among unmeasured patients was likewise similar across eras (4.3% vs 4.9%).

Assumptions and interpretation. This decomposition holds under the assumptions of direct standardization: that the stratum-specific incidences observed in Era 1 would have applied had Era 1 patients been monitored with the Era 2 surveillance distribution, and that measured and unmeasured patients within an era are exchangeable conditional on measurement status. Because receipt of POD5–7 testing was determined by clinical practice rather than by design — and is closely tied to discharge timing, since patients without a POD5–7 measurement were almost all discharged before POD5 — these assumptions cannot be verified in these data. Accordingly, the figure should be read as the proportion of the observed temporal rise that is explained by differences in surveillance intensity under the assumptions of this analysis, and not as a causal decomposition of the rise into detection and biology.

CI, confidence interval; POD, postoperative day.

### 3.4 Sodium trajectories and timing of the biological nadir

In the surveillance-complete subgroup, mean serum sodium trajectories differed clearly according to the subsequent development of delayed postoperative hyponatremia.

Patients who later developed delayed hyponatremia had slightly lower preoperative serum sodium concentrations than those who remained normonatremic (138.2 vs. 138.9 mmol/L). Although both groups demonstrated a similar early postoperative increase in serum sodium, patients who subsequently developed delayed hyponatremia showed a progressive decline beginning on POD3, reaching a mean nadir between POD6 and POD7 (approximately 131.8 mmol/L). In contrast, serum sodium remained stable at approximately 139 mmol/L throughout the postoperative course in patients without delayed hyponatremia.

The timing of the first recorded hyponatremic event was strongly influenced by surveillance frequency. Among the 46 patients with delayed postoperative hyponatremia, the first documented serum sodium concentration below 135 mmol/L appeared most frequently on POD3 (26.1%) and POD4 (21.7%), followed by POD5 (15.2%), POD6 (17.4%), POD7 (10.9%), and POD8–10 (8.7%).

However, this distribution closely mirrored the availability of sodium measurements rather than the biological timing of hyponatremia. Serum sodium was measured in all patients on POD3 and in 92.5% on POD4, whereas measurements were available in only 35.6%, 25.3%, and 14.2% of patients on POD5, POD6, and POD7, respectively. Therefore, the apparent peak on POD3–4 primarily reflects measurement frequency rather than the true biological onset of delayed hyponatremia.

The biological timing of sodium nadir was more accurately represented in the surveillance-complete subgroup, where repeated measurements allowed identification of each patient’s lowest postoperative sodium concentration (**Figure 2**). Among the 38 patients with delayed postoperative hyponatremia in this subgroup, sodium nadir occurred on POD3 in 7.9%, POD4 in 21.1%, POD5 in 15.8%, POD6 in 23.7%, POD7 in 21.1%, and POD8–10 in 10.5% of patients.

**Figure 2:**
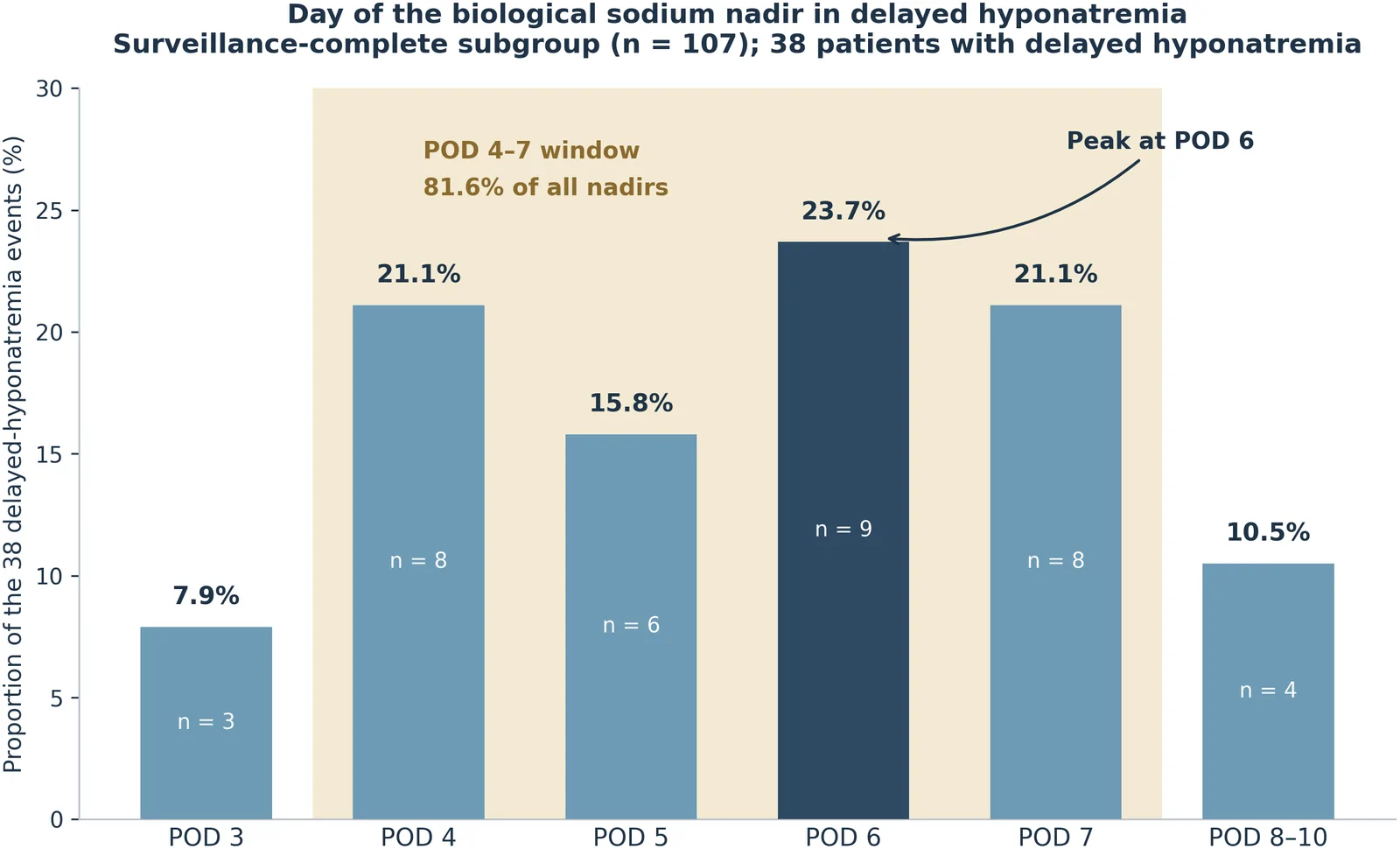
Day of the biological sodium nadir in delayed hyponatremia. Among the 38 patients with delayed hyponatremia in the surveillance-complete subgroup (n = 107), the sodium nadir clustered at POD 6 (24%), with approximately 82% of all nadirs occurring within the POD 4–7 window (shaded). Because routine inpatient sodium measurement becomes sparse after POD4 — most patients are discharged by then (median length of stay 4 days) — delayed hyponatremia arising in this window is frequently missed unless surveillance is extended into the outpatient period.

Overall, the biological nadir occurred approximately three days later than suggested by the first recorded event, peaked on POD6, and approximately 82% of all sodium nadirs occurred between POD4 and POD7. These findings are consistent with previous reports recommending postoperative sodium assessment during POD5–7 (6, 9, 15, 16) and provide direct quantitative support for routine sodium surveillance during this interval.

### 3.5 Early postoperative hyponatremia as a bedside predictor

Early postoperative hyponatremia, defined as any serum sodium concentration below 135 mmol/L during POD0–2, was observed in 32 of 281 patients (11.4%) in the primary cohort and in 18 of 107 patients (16.8%) in the surveillance-complete subgroup.

Among the individual bedside-available variables examined, early postoperative hyponatremia showed the largest univariable association with subsequent delayed postoperative hyponatremia.

In the primary cohort, delayed hyponatremia developed in 15 of 32 patients (46.9%) with early postoperative hyponatremia compared with 31 of 249 patients (12.4%) without early hyponatremia, corresponding to a relative risk of 3.77 (95% CI, 2.30–6.17) and a univariable Firth odds ratio of 6.14 (95% CI, 2.79–13.52; *p* < 0.001).

In the surveillance-complete subgroup, delayed postoperative hyponatremia occurred in 11 of 18 patients (61.1%) with early postoperative hyponatremia compared with 27 of 89 patients (30.3%) without early hyponatremia (relative risk, 2.01).

A biphasic sodium trajectory, characterized by both early and delayed postoperative hyponatremia, was observed in 15 patients (5.3%), whereas 17 patients (6.0%) experienced early hyponatremia only and 31 patients (11.0%) developed isolated delayed postoperative hyponatremia.

Taken together, these findings indicate that early postoperative hyponatremia identifies approximately half of all patients who subsequently develop delayed postoperative hyponatremia, and that it does so while selecting a subgroup of manageable size. Whether selecting patients in this way improves outcomes was not assessed.

### 3.6 Comparison of biphasic and other postoperative sodium trajectories

Patients were classified into four mutually exclusive postoperative sodium trajectory groups: no hyponatremia (n = 218, 77.6%), early-only hyponatremia (n = 17, 6.0%), delayed-only hyponatremia (n = 31, 11.0%), and biphasic hyponatremia (n = 15, 5.3%) (**Supplementary Table 2**).

Among the 32 patients with early postoperative hyponatremia, 15 (46.9%) subsequently developed delayed postoperative hyponatremia, whereas 17 (53.1%) did not. To identify factors associated with progression to a biphasic course, patients with biphasic and early-only hyponatremia were compared.

Age, sex, preoperative serum sodium concentration, early postoperative sodium nadir, and the magnitude of the early postoperative sodium decline were comparable between the two groups (all p > 0.30). Previous pituitary surgery, however, was more common among patients with a biphasic course (26.7% vs. 0%; Fisher’s exact p = 0.038).

Patients with a biphasic pattern also underwent more intensive postoperative sodium surveillance, with a median of five sodium measurements during POD3–10 compared with two measurements in the early-only group (Mann–Whitney p = 0.004). Likewise, at least one sodium measurement during POD5–7 was available in 73% and 41% of patients, respectively.

Among patients with early postoperative hyponatremia, Firth penalized logistic regression identified the number of postoperative sodium measurements as the only independent predictor of a recorded biphasic course (OR per additional measurement, 1.91; 95% CI, 1.15–3.18; p = 0.013).

Within the surveillance-complete subgroup, 61.1% of patients with early postoperative hyponatremia developed a biphasic pattern, compared with only 28.6% among those without POD5–7 sodium measurements. This finding suggests that the lower frequency of biphasic cases observed in the overall cohort primarily reflects incomplete detection of delayed hyponatremia rather than true biological differences.

Although patients with a biphasic course tended to have more extensive tumor characteristics, including suprasellar extension, optic chiasm compression, and cavernous sinus invasion, none of these comparisons reached statistical significance. Importantly, the severity of delayed hyponatremia was similar in patients with biphasic and delayed-only trajectories, indicating that the biphasic pattern reflects differences in timing rather than biochemical severity.

### 3.7 Other clinical associations

In univariable Firth regression analyses (**Table 1**), female sex was significantly associated with delayed postoperative hyponatremia (OR, 2.57; 95% CI, 1.34–4.91; p = 0.004).

Lower preoperative serum sodium concentration (OR per mmol/L, 0.88; p = 0.033), supra- or extrasellar extension (OR, 2.39; p = 0.012), optic chiasm compression (OR, 1.97; p = 0.044), and year of surgery (OR per 5 years, 1.51; 95% CI, 1.17–1.96; p = 0.002) were also associated with delayed postoperative hyponatremia.

In contrast, cavernous sinus invasion, previous pituitary surgery, previous Gamma Knife radiosurgery or radiotherapy, and functional adenoma status were not significantly associated with the outcome.

Histopathological subtype-specific event rates ranged from 10.7% (3/28) in corticotroph adenomas to 25.0% (9/36) in lactotroph adenomas. However, these subgroup analyses were considered descriptive because each subgroup contained relatively few events.

Although supra- or extrasellar extension was associated with delayed postoperative hyponatremia, none of the continuous tumor size measurements—including craniocaudal, anteroposterior, transverse diameter, or estimated ellipsoid tumor volume—showed significant associations. These findings suggest that anatomical extension rather than absolute tumor size may be more relevant to postoperative sodium disturbances. This anatomical association is concordant with a previous single-institution study of non-functioning pituitary adenomas, which identified larger craniocaudal tumor extension as an independent predictor of delayed postoperative hyponatremia (17).

Because the apparent effect of surgical year disappeared after accounting for surveillance intensity, calendar period was not retained in the primary multivariable model.

### 3.8 Age-by-sex interaction

Age was not associated with delayed postoperative hyponatremia in the overall cohort (OR per 10 years, 1.11; 95% CI, 0.90–1.37; p = 0.35). However, formal interaction testing demonstrated a significant interaction between age and sex (likelihood ratio test, p = 0.010).

Among women, age was not associated with delayed postoperative hyponatremia (OR per 10 years, 0.97; 95% CI, 0.75–1.27; p = 0.83), and age alone showed no discriminatory ability (AUC = 0.50).

In contrast, age was independently associated with delayed postoperative hyponatremia among men (OR per 10 years, 1.49; 95% CI, 1.04–2.13; p = 0.030), with moderate discrimination (AUC = 0.65).

Delayed postoperative hyponatremia remained consistently frequent across all female age groups, whereas the incidence increased progressively with age in men. Using the Youden index, 48 years was identified as the age threshold that maximized separation among male patients within this dataset. Men younger than 48 years had an incidence of 6.3%, compared with 17.2% among those aged 48 years or older, corresponding to a 2.7-fold relative increase. Age was modeled as a continuous variable in every regression analysis reported in this study; this threshold was not entered into any model and is presented only as an exploratory illustration of the age gradient observed in men.

These findings indicate that female sex represents a relatively constant risk factor irrespective of age, whereas increasing age further stratifies risk among male patients.

### 3.9 Sex-stratified early sodium threshold and composite triage strategy

Because both female sex and early postoperative hyponatremia were independently associated with delayed postoperative hyponatremia, we evaluated whether early sodium thresholds differed according to sex and whether combining these variables improved clinical risk stratification.

The predictive performance of the early postoperative sodium nadir differed substantially between sexes. Among men, the early sodium nadir demonstrated good discrimination (AUC = 0.720), whereas discrimination was only modest among women (AUC = 0.588).

Using the conventional threshold of <135 mmol/L, early postoperative hyponatremia demonstrated high specificity among men (0.94) with a positive likelihood ratio of 7.31. Among women, specificity remained high (0.90), although the positive likelihood ratio was lower (2.85). Lowering the threshold to <134 mmol/L modestly increased specificity in men but substantially reduced sensitivity. Therefore, the standard threshold of 135 mmol/L remained the most clinically appropriate cutoff.

Risk stratification based on sex and early postoperative hyponatremia revealed distinct patterns. Among men, delayed postoperative hyponatremia increased from 6.9% in patients without early hyponatremia to 46.7% in those with early hyponatremia. Among women, the corresponding increase was from 20.0% to 47.1%.

Several practical triage strategies were evaluated. A simple rule based on female sex or early postoperative hyponatremia identified 49% of the cohort while capturing 78% of all delayed postoperative hyponatremia events and achieving a negative predictive value of 93%.

A more sensitive strategy, defined as all women plus men above the data-derived age cutpoint or men with POD0–2 serum sodium <135 mmol/L, identified 68% of patients, captured 94% of all delayed hyponatremia events, and achieved a negative predictive value of 97%. Because the age cutpoint was chosen to maximize separation in these same data, this figure is an optimistic estimate of what the rule would achieve elsewhere.

Conversely, restricting surveillance to patients with POD0–2 serum sodium <134 mmol/L identified only 4% of the cohort and missed most delayed hyponatremia events despite its high positive predictive value.

These strategies are presented as exploratory illustrations of how sex, age, and early sodium distribute risk in this cohort rather than as validated triage rules. Their operating characteristics were estimated in the same data from which they were derived and are therefore optimistic. One such illustration would direct POD5–7 sodium measurement to all female patients and to men who are older or who have early postoperative hyponatremia. Whether younger men without early postoperative hyponatremia could forgo scheduled biochemical surveillance is a question these data raise but cannot answer: no clinical outcomes were recorded, the strategies were derived and evaluated in the same dataset, and the safety of omitting testing in any group would have to be established prospectively before it could be recommended.

### 3.10 Multivariable analyses

Model A, the primary model for the secondary risk-stratification objective, was developed in the surveillance-complete subgroup (105 complete cases with 36 events) and included female sex, preoperative serum sodium concentration, early postoperative hyponatremia, and functional adenoma status.

Female sex remained independently associated with delayed postoperative hyponatremia (OR, 2.73; 95% CI, 1.17–6.39; p = 0.021). Early postoperative hyponatremia also retained a positive association (OR, 2.83; 95% CI, 0.86–9.33), although this did not reach conventional statistical significance after multivariable adjustment (p = 0.088). Neither preoperative serum sodium concentration (OR per mmol/L, 0.97; p = 0.71) nor functional adenoma status (OR, 1.02; p = 0.96) remained independently associated with the outcome.

Model B, which replaced baseline sodium with the change in serum sodium from the preoperative value to POD2, produced similar results. Female sex remained an independent predictor, whereas the magnitude of the early postoperative sodium decline did not provide additional predictive information beyond early postoperative hyponatremia.

As a sensitivity analysis, Model C was fitted in the entire cohort and included an indicator for POD5–7 sodium surveillance. After adjustment for sex, preoperative sodium concentration, and early postoperative hyponatremia, the presence of late sodium surveillance carried the largest adjusted association with recorded delayed postoperative hyponatremia (adjusted OR, 11.05; 95% CI, 4.68–26.09; p < 0.001). Although this estimate reflects the upper limit of surveillance-related ascertainment bias by definition, it substantially exceeded the effect size of any biological predictor and quantitatively supports the primary hypothesis of this study.

Complete model coefficients are provided in **Supplementary Table 1**.

### 3.11 Model performance and clinical utility

In the surveillance-complete subgroup, Model A achieved an apparent area under the receiver operating characteristic curve (AUC) of 0.693 (95% CI, 0.582–0.802).

After internal validation using 2,000 bootstrap resamples, the optimism-corrected AUC was 0.645. Calibration was acceptable, with an apparent calibration slope of 1.02 and an optimism-corrected slope of 0.77, indicating only moderate model optimism.

The apparent Brier score was 0.204, increasing to 0.224 after optimism correction, which was essentially identical to the null-model Brier score (0.225). These findings indicate that, although the model provides useful risk stratification at the population level, its ability to estimate individual patient risk remains modest.

Decision curve analysis demonstrated a positive net clinical benefit across threshold probabilities of approximately 20%–50%, the range considered most relevant for selecting patients for targeted outpatient sodium surveillance. At a 30% probability threshold, the model achieved a sensitivity of 75%, specificity of 58%, a positive predictive value of 48%, and a negative predictive value of 82%.

Overall, these findings support the model as an exploratory risk-stratification approach that may help prioritize outpatient sodium monitoring at the group level. With an optimism-corrected AUC of 0.645 and an optimism-corrected Brier score essentially equal to that of the null model, it is not a stand-alone individualized risk calculator and is not ready for clinical deployment.

### 3.12 Sensitivity analyses

Severe delayed postoperative hyponatremia (serum sodium <130 mmol/L) occurred in 17 of 281 patients (6.0%), whereas profound hyponatremia (serum sodium <125 mmol/L) occurred in seven patients (2.5%).

The direction of association for female sex and early postoperative hyponatremia remained unchanged when these more stringent outcome definitions were applied, although the limited number of events precluded reliable multivariable analyses.

Excluding patients with preoperative hyponatremia (n = 12) left the apparent incidence of delayed postoperative hyponatremia essentially unchanged at 16.4% (44/269) and did not materially alter the observed associations.

Similarly, exclusion of patients with previous pituitary surgery or previous radiotherapy yielded comparable results. Excluding the five patients with non-adenomatous histopathology described in Section 2.1 left 276 patients with 45 events (16.3%) in the primary cohort and 103 patients with 37 events (35.9%) in the surveillance-complete subgroup, and did not materially change the multivariable estimates (female sex: OR, 2.76; p = 0.021; early postoperative hyponatremia: OR, 2.87; p = 0.086).

When analyses were restricted to patients treated after 2016, the apparent incidence of delayed postoperative hyponatremia increased to 24.8% (28/113). However, within the corresponding surveillance-complete subgroup, the incidence remained similar (39.6%), further supporting the influence of surveillance intensity on the observed incidence.

### 3.13 Length of hospital stay

The median length of hospital stay was 4 days (interquartile range [IQR], 4–5) among patients without delayed postoperative hyponatremia and 6 days (IQR, 5–10) among those who developed delayed postoperative hyponatremia (Mann–Whitney p < 10⁻¹²).

This association should be interpreted cautiously because it is inherently bidirectional. Longer hospitalization increases the opportunity to detect delayed hyponatremia, whereas delayed hyponatremia itself may prolong hospital stay. Consequently, length of stay was considered a descriptive outcome rather than a predictor and was not included in the multivariable prediction models.

## 4 Discussion

In this 21-year single-center cohort of patients undergoing microscopic transsphenoidal surgery by a single surgical team, we found that the apparent incidence of delayed postoperative hyponatremia varied more than threefold according to the intensity of postoperative sodium surveillance. Patients who underwent systematic sodium assessment during POD5–7 had substantially higher recorded event rates than those without late postoperative measurements. These findings suggest that much of the wide variation in reported incidence across previous studies is likely attributable to differences in surveillance strategies rather than true biological variation. In addition, early postoperative hyponatremia identified a subgroup with a higher observed event rate before hospital discharge, which may merit evaluation as a criterion for targeted outpatient sodium surveillance in prospective studies.

### 4.1 Surveillance intensity and the reported incidence

One of the principal findings of this study is that postoperative sodium surveillance substantially influences the recorded incidence of delayed postoperative hyponatremia. Although previous studies have reported incidence rates ranging from approximately 5% to 30%, this variability has generally been attributed to differences in patient characteristics, surgical technique, or perioperative management (2–11). Our findings suggest that differences in surveillance intensity may account for a considerable proportion of this heterogeneity.

This conclusion was supported by several complementary analyses. First, patients who underwent sodium measurement during POD5–7 had a markedly higher recorded incidence of delayed postoperative hyponatremia than those without late surveillance. Second, the apparent increase in incidence over time largely disappeared after restricting the analysis to patients with comparable postoperative surveillance. Finally, under the assumptions of the direct-standardization analysis, approximately 83% of the observed temporal increase in delayed postoperative hyponatremia was explained by differences in surveillance intensity (**Table 3**), with the remainder unexplained by surveillance. Because those assumptions cannot be verified in observational data, this partition quantifies how much of the temporal rise is accounted for by measurement under a stated model, and is not a causal decomposition into detection and biology.

Collectively, these findings indicate that comparisons of delayed postoperative hyponatremia across institutions or time periods should be interpreted cautiously unless postoperative sodium surveillance is comparable.

A potential objection to the surveillance interpretation is that non-measurement at POD5–7 is not random but largely a consequence of discharge before that window: patients without a POD5–7 sodium had a markedly shorter length of stay (median 4 vs. 6 days) and 99% had been discharged before POD5. This is informative missingness, and we cannot fully exclude that patients fit for early discharge were at genuinely lower biological risk. Two observations argue against this explaining the apparent secular rise. First, the biological sodium nadir clustered around POD6 (**Figure 2**), so patients discharged on POD4 leave precisely before the event window opens; events occurring at home are captured only if they prompt readmission, and delayed hyponatremia is the single most common cause of unplanned readmission after transsphenoidal surgery (1). Second, and most directly, when monitoring was held constant by restricting to the surveillance-complete subgroup, the incidence was essentially identical across operative eras (33.3% vs. 36.1%, Fisher p = 1.00; **Table 2**), whereas the apparent whole-cohort incidence doubled (10.3% to 20.7%) in parallel with a rise in POD5–7 measurement from 21% to 51% of patients. This expansion of monitoring coincides with the marked growth of the delayed-hyponatremia literature and of clinical awareness from approximately 2014–2016 onward, and a meta-analysis found no variation in pooled incidence by publication year (5). We therefore interpret the apparent temporal increase as predominantly an ascertainment phenomenon driven by intensified postoperative sodium surveillance rather than a change in the underlying biology, while explicitly acknowledging that the cross-sectional measured-versus-unmeasured contrast is confounded by discharge timing.

### 4.2 Early postoperative hyponatremia as a bedside marker

Our study also identified early postoperative hyponatremia during POD0–2 as a bedside marker of subsequent delayed postoperative hyponatremia. We do not describe it as the strongest available predictor, because the strength of the supporting evidence differs across analyses in a way that should be stated precisely. On univariable analysis in the primary cohort the association was strong and statistically significant (OR, 6.14; 95% CI, 2.79–13.52; p < 0.001), and it persisted after multivariable adjustment when the full cohort was analyzed with an explicit surveillance indicator (Model C: adjusted OR, 4.81; 95% CI, 1.81–12.79; p = 0.002). Within the smaller surveillance-complete subgroup the adjusted point estimate remained elevated and in the same direction, but its confidence interval included unity (Model A: OR, 2.83; 95% CI, 0.86–9.33; p = 0.088). Only 18 patients in that subgroup had early postoperative hyponatremia, so this result is more readily interpreted as limited precision than as absence of an association; nonetheless, we do not claim independent statistical significance for this predictor in the primary model. Early postoperative hyponatremia is accordingly described here as a sentinel marker requiring prospective confirmation rather than as an independently validated predictor. Nearly one-half of patients with early hyponatremia later developed delayed hyponatremia, and this proportion exceeded 60% in patients who underwent systematic late sodium surveillance.

Unlike several previously proposed prediction models, which rely on variables obtained relatively late after surgery or on laboratory findings that overlap with the outcome itself, early postoperative sodium measurements are routinely available before hospital discharge in most centers. Consequently, this predictor can be incorporated into routine postoperative care without additional investigations or resource utilization.

Rather than supporting universal outpatient sodium testing, our findings favor a risk-adapted strategy in which patients with early postoperative hyponatremia undergo targeted serum sodium assessment during POD5–7, when delayed hyponatremia is most likely to occur.

### 4.3 Sex differences and the age-by-sex interaction

Female sex was independently associated with delayed postoperative hyponatremia across both univariable and multivariable analyses, consistent with several previous reports (1, 3). Although the underlying mechanisms remain incompletely understood, sex-related differences in vasopressin sensitivity, renal water handling, and body water composition have been proposed as potential contributors (10). Because physiological measurements relevant to these mechanisms were not available in the present study, our findings should be interpreted as identifying a reproducible clinical association rather than establishing causality.

An additional finding was the significant interaction between age and sex. Among women, the risk of delayed postoperative hyponatremia remained relatively constant across all age groups. In contrast, risk increased progressively with age among men. A cutpoint of 48 years maximized separation within this cohort, but it was derived from these data by the Youden index, was not entered into any model, and should not be read as a validated threshold for risk stratification. Although this observation requires external validation, it suggests that postoperative surveillance strategies may benefit from considering age differently in men and women. Female sex alone identified a consistently high-risk population, whereas age provided additional discrimination primarily among male patients.

The predictive value of early postoperative sodium also differed according to sex. Early hyponatremia demonstrated better discrimination among men than women, although the conventional threshold of 135 mmol/L remained clinically appropriate in both groups. These findings suggest that sex-specific risk profiles may improve postoperative surveillance strategies and should be explored in future validation studies.

### 4.4 Tumor extension and anatomical risk markers

Among tumor-related factors, supra- or extrasellar extension and optic chiasm compression were each associated with delayed postoperative hyponatremia on univariable analysis (odds ratios 2.39 and 1.97, respectively), whereas no continuous measure of tumor size was. This pattern—an association carried by the anatomical fact of superior extension rather than by tumor volume—is concordant with a previous single-institution study that identified larger craniocaudal tumor extension, together with greater preoperative pituitary-stalk deviation, as an independent predictor of delayed hyponatremia (17). These observations are most consistent with a mechanism in which superior tumor growth and the traction or distortion it imposes on the pituitary stalk and posterior pituitary, rather than tumor size per se, promote dysregulated vasopressin release. Because these anatomical markers are available preoperatively, they may complement the early-sodium sentinel as an additional, imaging-based indicator of patients warranting closer POD5–7 surveillance.

### 4.5 Clinical implications

The present findings have direct implications for postoperative follow-up after transsphenoidal pituitary surgery.

Routine sodium surveillance for every patient after discharge may not be practical in many institutions. Conversely, relying solely on inpatient sodium measurements obtained during the first postoperative days is likely to underestimate the true incidence of delayed postoperative hyponatremia because the biological sodium nadir generally occurs several days later.

Our results generate a hypothesis about targeted surveillance rather than establishing one. Patients with early postoperative hyponatremia, together with women and older male patients, had higher observed event rates in this cohort, which raises the possibility that scheduled outpatient sodium assessment during POD5–7 could be concentrated on them. Whether such an approach would improve detection without missing clinically important events, and whether reducing testing in lower-risk patients is safe, cannot be determined from these data: no clinical outcomes were available, and the strategies were derived and evaluated in the same cohort.

Although the proposed triage strategy requires external validation before routine implementation, it offers a simple framework based entirely on information available before hospital discharge.

### 4.6 Strengths and limitations

This study has several strengths.

First, all operations were performed by the same experienced pituitary surgery team using a uniform microscopic transsphenoidal technique over a 21-year period, minimizing variability related to surgical practice. Second, the primary outcome was reconstructed directly from raw laboratory measurements rather than predefined database variables, reducing outcome misclassification. Third, surveillance intensity was explicitly quantified and incorporated into the analyses, allowing estimation of its contribution to the apparent incidence of delayed postoperative hyponatremia. Finally, prediction models were internally validated using bootstrap resampling, providing optimism-corrected estimates of model performance.

Several limitations should also be acknowledged.

The retrospective single-center design limits the generalizability of the findings. Late postoperative sodium measurements were not obtained systematically in all patients, and surveillance intensity itself was partly determined by clinical practice. Although this characteristic formed the basis of the primary study hypothesis, it also introduces the possibility of informative observation bias. A further consideration concerns the length of the study period. Although the operating team and the microscopic transsphenoidal technique were constant across 21 years, many other components of perioperative care were not, and were not recorded in a form that permitted adjustment. Postoperative fluid management, glucocorticoid replacement practice, management of diabetes insipidus, discharge criteria, access to outpatient laboratory testing, the intensity and structure of endocrine follow-up, referral patterns and patient selection, readmission thresholds, and patient education about fluid intake may all have changed over two decades. Any of these could plausibly influence either the occurrence of delayed hyponatremia or the probability that it is recorded. The temporal comparisons reported here may therefore reflect secular changes in care beyond surveillance intensity, and the surveillance-standardized analysis should not be read as having removed all time-related confounding. Relatedly, the multivariable prediction model was developed in the surveillance-complete subgroup rather than in the full primary cohort. That subgroup was assembled through clinician-initiated sampling rather than by protocol, so it is more intensively monitored than the operated population as a whole and cannot be assumed to represent it; the model coefficients and performance estimates should therefore be read as applying to completely ascertained patients, and their transportability to unselected postoperative cohorts remains untested. The 48-year age threshold reported among men was likewise derived from these data using the Youden index rather than from any external biological or clinical standard. Cutpoints obtained in this way are optimized to the sample in which they are estimated, are typically unstable when re-estimated in other datasets, and commonly shift or lose discrimination on external application; the threshold should accordingly be regarded as a descriptive illustration of the age gradient in men and requires external validation before any clinical use. In addition, serum and urine osmolality, urine sodium concentration, volume status, and detailed endocrine data were not consistently available throughout the study period. Consequently, delayed hyponatremia could not be classified according to its underlying pathophysiological mechanism. More importantly, clinical outcome data were not available. The dataset does not record unplanned readmission, emergency department presentation, neurological symptoms, seizures or altered mental status, treatment administered for hyponatremia, fluid restriction, hypertonic saline use, or intensive care admission. This study therefore evaluates the biochemical detection of delayed hyponatremia and not its clinical consequences, and it cannot address whether more intensive surveillance improves patient outcomes. The distinction matters directly for how our findings should be applied: identifying additional, largely mild biochemical hyponatremia through expanded testing is not equivalent to preventing harm, and a targeted surveillance strategy of the kind suggested here requires prospective evaluation against clinical endpoints before its benefit can be assumed. Finally, the proposed prediction model underwent internal but not external validation; therefore, its clinical applicability should be confirmed in independent cohorts before widespread adoption.

## 5 Conclusions

In this homogeneous cohort of patients undergoing microscopic transsphenoidal pituitary surgery, the apparent incidence of delayed postoperative hyponatremia varied more than threefold according to the intensity of postoperative sodium surveillance. These findings suggest that a substantial proportion of the heterogeneity reported across previous studies may reflect differences in how the outcome was ascertained rather than differences in the underlying event rate; because surveillance was determined by clinical practice rather than by design, this remains an inference from an observational comparison.

As a secondary and exploratory finding, early postoperative hyponatremia represents a simple bedside marker that may identify patients at increased risk before hospital discharge. These data raise, but do not settle, the possibility that a postoperative surveillance strategy informed by early sodium measurements, sex, and age could concentrate testing where events are most frequent. Because clinical outcomes were not recorded and the strategies were not validated outside this cohort, neither their benefit nor the safety of reducing testing in lower-risk patients can be claimed here. This risk-stratification component achieved only modest discrimination, was developed in a clinician-selected subgroup, and has not been externally validated; it is offered as a hypothesis for prospective evaluation and not as a tool for immediate clinical use.

Because this study evaluated biochemical detection rather than clinical outcome, it cannot establish that more intensive surveillance benefits patients. Prospective multicenter studies with standardized postoperative sodium surveillance and prospectively collected clinical endpoints — readmission, symptomatic hyponatremia, and treatment requirements — are needed to validate these findings and to determine the optimal surveillance strategy after transsphenoidal pituitary surgery.

## Data Availability

The de-identified dataset and analysis code that support the findings of this study are available from the corresponding author on reasonable request, subject to local data-protection regulations.
