# Supplementary material for: Surveillance intensity influences the observed incidence of delayed hyponatremia after microscopic transsphenoidal pituitary surgery: early sodium and sex as risk stratifiers
Source: Front Endocrinol (Lausanne). 2026 Sep 24;17:1946832. doi: 10.3389/fendo.2026.1946832 (PMC13645545; doi:10.3389/fendo.2026.1946832)
Supplement: Supplementary file 1 [file SupplementaryFile1.docx]

**Supplementary Material**

*Surveillance Intensity Influences the Observed Incidence of Delayed Hyponatremia After Microscopic Transsphenoidal Pituitary Surgery: Early Sodium and Sex as Risk Stratifiers*

*This document contains, in order: Supplementary Methods (Sections S1–S8); Supplementary Table S1; Supplementary Table S2; the completed STROBE and TRIPOD reporting checklists; and the full specification of the prediction model.*

**Supplementary Methods**

*Operational definitions, estimation procedures, and resampling protocol*

This appendix documents the analysis in sufficient detail to permit independent replication from the source laboratory data. It is intended to complement, not replace, the description in the Materials and Methods section of the main text.

**S1. Software environment**

All analyses were performed in Python 3.12 with pandas 2.2, NumPy 2.3, SciPy 1.17, and scikit-learn 1.6. Firth penalized logistic regression was implemented directly rather than through a package, as described in S3. All random processes were seeded; the seed used for the reported results is stated in S5. Re-running the pipeline with the stated seed reproduces every figure reported in the manuscript exactly.

**S2. Operational variable definitions**

Serum sodium was extracted as eleven separate raw fields: the day-of-surgery value and one value for each of postoperative days (POD) 1 through 10. All eleven were coerced to numeric, with non-numeric entries treated as missing. No sodium value was imputed at any point in the analysis. Variables were then derived as follows.

**Delayed postoperative hyponatremia (primary outcome).** True if any non-missing sodium value among POD3 through POD10 was below 135 mmol/L; otherwise false. The outcome was derived exclusively from these raw laboratory fields and not from any pre-existing database flag. During verification the pre-existing flag disagreed with the laboratory-derived definition in 20 patients, and the laboratory-derived definition was retained throughout.

**Early postoperative hyponatremia.** True if any non-missing sodium value among the day-of-surgery, POD1, and POD2 fields was below 135 mmol/L.

**Primary analysis cohort.** Patients with at least one non-missing sodium value among POD3 through POD10 (n = 281 of 285).

**Surveillance-complete subgroup.** Patients with at least one non-missing sodium value among POD5, POD6, and POD7 (n = 107).

**Surveillance flag (Model C covariate).** Binary indicator equal to 1 for membership in the surveillance-complete subgroup.

**Number of late measurements.** Count of non-missing sodium values among POD3 through POD10.

**Sex.** Coded in the source file as 0 for female and 1 for male; the analysis variable is female sex (1 = female). This coding is stated explicitly because the source coding is the reverse of the conventional direction.

**Functional adenoma proxy.** True for corticotroph, somatotroph, lactotroph, and plurihormonal/mixed lineage; false for gonadotroph and non-functioning adenomas. This variable was derived from the histopathological diagnosis field after whitespace trimming and case normalization. A separate pre-existing column in the source file labelled functioning adenoma classifies gonadotroph adenomas as functioning and was not used.

**Operative year.** Parsed from the surgical date field, which contains a mixture of native date values and text-formatted dates. One entry with the evident typographical year 1010 was corrected to 2010 by reference to its sequential record number and to adjacent surgical dates.

**Sodium trajectory.** Four mutually exclusive categories defined by the cross-classification of early and delayed hyponatremia: neither, early only, delayed only, and biphasic (both).

**S3. Firth penalized logistic regression**

Firth's method removes the first-order small-sample bias of the maximum-likelihood estimator by adding the Jeffreys invariant prior as a penalty, equivalently by modifying the score equation with the leverage-weighted term shown below. It also guarantees finite estimates under complete or quasi-complete separation. The modified score equation solved at each iteration is

U(b) = X' ( y - p + h * (0.5 - p) )

where p is the vector of fitted probabilities, X the design matrix including the intercept column, and h the diagonal of the hat matrix H = W^(1/2) X (X' W X)^(-1) X' W^(1/2), with W = diag(p(1-p)). Coefficients were obtained by Newton–Raphson iteration:

b <- b + (X' W X)^(-1) U(b)

**Implementation details.** Coefficients were initialized at zero. The information matrix was inverted directly, falling back to the Moore–Penrose pseudoinverse if the direct inversion failed. Step halving was applied to bound the magnitude of any single Newton–Raphson step. Iteration terminated when the maximum absolute coefficient change fell below 1e-8, subject to a maximum of 200 iterations; all reported models converged well within this limit. Standard errors were taken as the square roots of the diagonal of the inverse information matrix evaluated at the converged estimate, and p-values from the corresponding Wald statistic referred to the standard normal distribution. Confidence intervals are Wald intervals on the log-odds scale, exponentiated. The implementation was verified against published worked examples before use.

Firth penalization was applied to all univariable and multivariable models by design, and was not introduced selectively for models exhibiting separation.

**S4. Model specification, events per variable, and complete cases**

Three multivariable models were specified in advance. Covariate counts were fixed a priori; no covariate was added, removed, or reordered on the basis of statistical significance or model performance, and no automated selection procedure was used at any stage.

Model A (surveillance-complete subgroup): female sex, preoperative sodium, early hyponatremia, functional adenoma proxy. Complete cases 105, events 36, covariates 4, events per variable (EPV) 9.0.

Model B (surveillance-complete subgroup): female sex, preoperative-to-POD2 sodium change, early hyponatremia. Complete cases 105, events 36, covariates 3, EPV 12.0.

Model C (full primary cohort): female sex, preoperative sodium, early hyponatremia, surveillance flag. Complete cases 275, events 44, covariates 4, EPV 11.0.

Complete-case analysis was used for preoperative sodium, the only covariate with missing values; two patients were excluded from the surveillance-complete models (107 to 105) and six from the full-cohort model (281 to 275). Missingness of late sodium measurements was treated as informative, was not imputed under any missing-at-random assumption, and was instead analyzed as an exposure through the surveillance flag.

**S5. Discrimination, calibration, and internal validation**

Apparent discrimination was the area under the receiver operating characteristic curve computed from the fitted probabilities of the model refitted on the full analysis sample. The percentile confidence interval was obtained by drawing 2,000 bootstrap resamples of the analysis sample and evaluating the fixed apparent predictions within each resample.

Optimism correction is a separate procedure and follows Harrell's bootstrap protocol. For each of 2,000 resamples drawn with replacement from the analysis sample, the model was refitted from scratch within the resample; the performance of that refitted model was evaluated both in the resample in which it was fitted and in the original sample; and the difference between the two was recorded as the optimism for that replicate. Optimism was averaged across replicates and subtracted from the apparent value. Both procedures use 2,000 resamples so that a single resampling scheme underlies all reported performance metrics.

```
for b in 1..2000:
    idx  <- sample(1..n, n, replace = TRUE)
    fit_b <- firth(X[idx], y[idx])
    opt_b <- perf(fit_b, X[idx], y[idx]) - perf(fit_b, X, y)
corrected <- apparent - mean(opt_b)
```

Resamples in which the outcome was constant, or in which the refit failed to converge, were discarded and not replaced; the number of valid replicates is reported alongside the results. The random seed used for the reported results was 20260820.

**Calibration.** The calibration slope is the coefficient obtained by regressing the outcome on the linear predictor with an intercept, fitted by the same penalized method. Its optimism was corrected by the identical bootstrap protocol. The Brier score was compared with the null Brier score defined as the marginal event rate multiplied by its complement.

**Decision-curve analysis.** Net benefit was computed across threshold probabilities from 5% to 70% and compared with the treat-all and treat-none strategies.

**S6. Direct standardization**

The apparent inter-era rise in recorded incidence was decomposed by direct standardization on surveillance status. Let i(m) and i(u) denote the incidences observed in Era 1 among measured and unmeasured patients, and let w denote the proportion measured in Era 2. The standardized Era 1 incidence is

i_std = w * i(m) + (1 - w) * i(u)

With i(m) = 33.3%, i(u) = 4.3%, and w = 0.506, this yields 19.0%. The apparent rise of 10.5 percentage points (10.3% to 20.7%) is thereby partitioned into 8.7 points explained by the change in surveillance distribution and 1.7 points not explained by it. The assumptions required for this partition, and the reasons they cannot be verified in these data, are stated in Table 3 and in the Limitations section of the main text.

**S7. Statistical testing conventions**

Group comparisons used the Mann–Whitney U test for continuous variables without an assumption of symmetry, the Student t-test where symmetry was reasonable, and the chi-squared or Fisher exact test for categorical variables, with Fisher's test used whenever any expected cell count was small. Reported odds ratios from two-by-two tables are conditional maximum-likelihood estimates from the exact test and therefore differ slightly from the corresponding penalized regression estimates; both are reported where relevant and are not interchangeable. All tests were two-sided with a significance threshold of 0.05. No correction for multiple comparisons was applied; p-values from secondary and subgroup analyses are interpreted as descriptive.

**S8. Analysis pipeline**

The analysis proceeds in a fixed order: derivation of variables from the raw laboratory fields; construction of the two analysis populations; descriptive and univariable analysis; multivariable model fitting; internal validation and decision-curve analysis; direct standardization; and generation of tables and figures. Each stage depends only on the outputs of the preceding stages, and no stage is conditional on results obtained later in the sequence. The de-identified dataset and the analysis code are available from the corresponding author on reasonable request, subject to local data-protection regulations.

**Supplementary Tables**

**TABLE S1 | Multivariable Firth penalized logistic regression models for delayed postoperative hyponatremia.**

| **Predictor** | **OR (95% CI)** | **p** | **EPV** |
| --- | --- | --- | --- |
| ***Model A — primary model for the secondary risk-stratification objective (surveillance-complete subgroup; n = 105, 36 events)*** | | | |
| **Female sex** | **2.73 (1.17–6.39)** | **0.021** | **9.0** |
| Preoperative serum Na (per mmol/L) | 0.97 (0.84–1.13) | 0.709 |  |
| Early hyponatremia (POD0–2 <135 mmol/L) | 2.83 (0.86–9.33) | 0.088 |  |
| Functional adenoma (proxy) | 1.02 (0.43–2.40) | 0.964 |  |
| ***Model B — sodium-trajectory variant (surveillance-complete subgroup; n = 105, 36 events)*** | | | |
| **Female sex** | **2.76 (1.18–6.45)** | **0.019** | **12.0** |
| Preoperative-to-POD2 Na decline (per mmol/L) | 1.02 (0.93–1.12) | 0.704 |  |
| Early hyponatremia (POD0–2 <135 mmol/L) | 2.92 (0.93–9.18) | 0.067 |  |
| ***Model C — sensitivity analysis (full primary cohort with surveillance flag; n = 275, 44 events)*** | | | |
| **Female sex** | **3.48 (1.62–7.49)** | **0.001** | **11.0** |
| Preoperative serum Na (per mmol/L) | 0.99 (0.86–1.13) | 0.842 |  |
| **Early hyponatremia (POD0–2 <135 mmol/L)** | **4.81 (1.81–12.79)** | **0.002** |  |
| **POD5–7 measurement available (surveillance flag)** | **11.05 (4.68–26.09)** | **<0.001** |  |

*Outcome: delayed postoperative hyponatremia, defined as any serum sodium <135 mmol/L between POD3 and POD10. All models were fitted by Firth penalized logistic regression; confidence intervals are Wald intervals on the log-odds scale. Bold denotes p < 0.05.*

*The three models were specified in advance and are all reported. They address different questions and were not treated as competing candidates: no model was selected, dropped, or reordered on the basis of discrimination, calibration, decision-curve performance, or the statistical significance of its coefficients, and no covariate was retained or removed on the basis of its p-value.*

*Models A and B were fitted on the surveillance-complete subgroup (≥1 POD5–7 sodium measurement); Model C on the full primary cohort with an added binary surveillance flag. All models use complete-case analysis on preoperative sodium: the surveillance-complete subgroup falls from 107 to 105 patients (2 events lost) and the full cohort from 281 to 275 patients (2 events lost). Events per variable (EPV) is shown once per model.*

*The surveillance flag in Model C is, by construction, an upper bound on the magnitude of surveillance-driven ascertainment rather than a biological covariate; it is included to quantify that magnitude, not to estimate a causal effect of testing.*

**TABLE S2 | Clinical characteristics by postoperative sodium-trajectory pattern (primary cohort, n = 281).**

| **Characteristic** | **No hyponatremia (n = 218)** | **Early-only (n = 17)** | **Isolated delayed (n = 31)** | **Biphasic (n = 15)** |
| --- | --- | --- | --- | --- |
| Proportion of cohort | 77.6% | 6.0% | 11.0% | 5.3% |
| Female sex | 39% | 53% | 68% | 53% |
| Age, median (years) | 43 | 43 | 44 | 46 |
| Suprasellar/extrasellar extension | 48% | 47% | 67% | 73% |
| Optic chiasm compression | 48% | 41% | 63% | 67% |
| Cavernous sinus invasion | 47% | 29% | 47% | 60% |
| Prior pituitary surgery | 16% | 0% | 10% | 27% |
| Delayed-period Na nadir, median (mmol/L) | — | — | 132.0 | 131.2 |
| Severe nadir (<130 mmol/L) | — | — | 39% | 33% |
| Profound nadir (<125 mmol/L) | — | — | 19% | 7% |

*Patterns are mutually exclusive and defined by the cross-classification of early hyponatremia (any POD0–2 sodium <135 mmol/L) and delayed hyponatremia (any POD3–10 sodium <135 mmol/L): neither, early only, isolated delayed, and biphasic.*

*Values are percentages of non-missing observations unless otherwise stated; denominators for tumor characteristics are reduced by incomplete radiological data in a small number of patients. Delayed-period nadir and severity are reported only for patterns that include a delayed event. Subgroup comparisons are exploratory and the associated p-values reported in Section 3.6 of the main text are descriptive.*

*POD, postoperative day.*

**Reporting checklists**

*Item descriptions in the two tables below are abbreviated in the authors' own words for readability; the authoritative wording is that of the published STROBE and TRIPOD statements (references 12 and 13 of the main manuscript). Section numbers refer to the revised manuscript.*

**STROBE checklist — cohort studies**

| **Item** | **Topic** | **What the item requires (abbreviated)** | **Reported in** |
| --- | --- | --- | --- |
| ***Title and abstract*** | | | |
| 1a | Title and abstract | State the study design in the title or abstract | Title; Abstract |
| 1b | Title and abstract | Give a balanced summary of what was done and found | Abstract |
| ***Introduction*** | | | |
| 2 | Background | Explain the scientific background and rationale | Section 1, paragraphs 1–3 |
| 3 | Objectives | State specific objectives, including any pre-specified hypotheses | Section 1, final paragraphs (one primary and two secondary questions) |
| ***Methods*** | | | |
| 4 | Study design | Present key elements of design early in the paper | Section 2.1 |
| 5 | Setting | Describe setting, locations, and relevant dates | Section 2.1 (single tertiary centre; January 2004 – March 2025) |
| 6a | Participants | Give eligibility criteria and sources and methods of selection | Section 2.1; Figure 1 |
| 6b | Participants | Matched studies: matching criteria and numbers | Not applicable (unmatched cohort) |
| 7 | Variables | Define outcomes, exposures, predictors, confounders and effect modifiers | Sections 2.2, 2.4; Supplementary Methods S2 |
| 8 | Data sources / measurement | Give sources of data and details of assessment methods for each variable | Sections 2.1, 2.2; Supplementary Methods S2 |
| 9 | Bias | Describe any efforts to address potential sources of bias | Sections 2.3, 2.5, 2.6 (surveillance-related ascertainment bias is the study exposure); Discussion, Limitations |
| 10 | Study size | Explain how the study size was arrived at | Section 2.1 (all consecutive eligible patients over 21 years); Section 3.1 |
| 11 | Quantitative variables | Explain how quantitative variables were handled and why any grouping was used | Sections 2.4, 2.6 (age modeled continuously throughout; calendar period grouped for description only) |
| 12a | Statistical methods | Describe methods including control for confounding | Sections 2.6, 2.7 |
| 12b | Statistical methods | Describe methods used to examine subgroups and interactions | Sections 2.6, 3.8, 3.9 |
| 12c | Statistical methods | Explain how missing data were addressed | Section 2.5; Supplementary Methods S4 |
| 12d | Statistical methods | Explain how loss to follow-up was addressed | Section 2.5 (missing late sodium treated as informative and analysed as an exposure, not imputed) |
| 12e | Statistical methods | Describe any sensitivity analyses | Sections 2.6, 3.12 |
| ***Results*** | | | |
| 13a | Participants | Report numbers at each stage of the study | Section 3.1; Figure 1 |
| 13b | Participants | Give reasons for non-participation at each stage | Section 3.1 (four patients without any POD3–10 sodium); Figure 1 |
| 13c | Participants | Consider use of a flow diagram | Figure 1 |
| 14a | Descriptive data | Give characteristics of participants and information on exposures and confounders | Section 3.1; Table 1 |
| 14b | Descriptive data | Indicate number of participants with missing data for each variable | Table 1 (denominators of non-missing shown); Section 2.5; Supplementary Table S1 |
| 14c | Descriptive data | Summarise follow-up time | Sections 2.2, 3.13 (outcome window POD3–10; length of stay reported) |
| 15 | Outcome data | Report numbers of outcome events over time | Sections 3.1, 3.2, 3.4; Tables 1–3; Figure 2 |
| 16a | Main results | Give unadjusted and adjusted estimates with confidence intervals and state confounders adjusted for | Tables 1, 3; Sections 3.7, 3.10; Supplementary Table S1 |
| 16b | Main results | Report category boundaries when continuous variables were categorized | Sections 2.4, 3.3 (calendar periods and eras); Section 3.8 (48-year threshold, descriptive only) |
| 16c | Main results | Consider translating relative risk into absolute risk | Sections 3.5, 3.9 (event rates and predictive values by risk stratum) |
| 17 | Other analyses | Report other analyses done, including subgroups, interactions and sensitivity analyses | Sections 3.6, 3.8, 3.9, 3.12 |
| ***Discussion*** | | | |
| 18 | Key results | Summarise key results with reference to study objectives | Section 4, opening paragraph |
| 19 | Limitations | Discuss limitations, sources of potential bias and imprecision | Discussion, Strengths and limitations |
| 20 | Interpretation | Give a cautious overall interpretation considering objectives, limitations and other evidence | Discussion; Conclusions |
| 21 | Generalisability | Discuss the external validity of the study results | Discussion, Strengths and limitations (single centre, microscopic technique, clinician-selected subgroup) |
| ***Other information*** | | | |
| 22 | Funding | Give the source of funding and the role of funders | Funding |

*Item 6b is not applicable because the cohort was not matched. Items 12d and 14c are addressed by the study design rather than by conventional follow-up methods: the outcome window is fixed at POD3–10 and incomplete late sodium measurement is treated as informative missingness and analysed as an exposure rather than as loss to follow-up.*

**TRIPOD checklist — model development**

| **Item** | **Topic** | **What the item requires (abbreviated)** | **Reported in** |
| --- | --- | --- | --- |
| ***Title and abstract*** | | | |
| 1 | Title | Identify the study as developing or validating a prediction model, the target population and the outcome | Title; Abstract |
| 2 | Abstract | Provide a structured summary of objectives, setting, participants, outcome, methods, results and conclusions | Abstract |
| ***Introduction*** | | | |
| 3a | Background | Explain the medical context and rationale, including reference to existing models | Section 1, paragraph 3 (limitations of existing models relying on late or outcome-overlapping variables) |
| 3b | Objectives | Specify the objectives, including whether the study describes development or validation | Section 1, final paragraphs (development only; no external validation) |
| ***Methods*** | | | |
| 4a | Source of data | Describe the study design or data source and state the rationale | Section 2.1 |
| 4b | Source of data | Specify the key study dates | Section 2.1 (January 2004 – March 2025) |
| 5a | Participants | Specify key elements of the study setting and locations | Section 2.1 |
| 5b | Participants | Describe eligibility criteria | Sections 2.1, 2.3; Figure 1 |
| 5c | Participants | Give details of any treatments received | Section 2.1 (uniform microscopic transsphenoidal technique, single team) |
| 6a | Outcome | Define the outcome to be predicted and how and when it was assessed | Section 2.2; Supplementary Methods S2 |
| 6b | Outcome | Report any actions to blind assessment of the outcome | Section 2.2 (outcome derived mechanically from raw laboratory values, not by adjudication, so assessment could not be influenced by predictor values) |
| 7a | Predictors | Define all predictors and how and when they were measured | Section 2.4; Supplementary Methods S2 |
| 7b | Predictors | Report any actions to blind predictor assessment | Section 2.4 (all predictors extracted from routine records recorded before the outcome window opened) |
| 8 | Sample size | Explain how the study size was arrived at | Section 2.1; Section 2.6 (all eligible patients; events per variable fixed a priori, EPV 9.0–12.0); Supplementary Methods S4 |
| 9 | Missing data | Describe how missing data were handled, with reasons and method | Section 2.5; Supplementary Methods S4 |
| 10a | Statistical analysis | Describe how predictors were handled in the analysis | Sections 2.4, 2.6; Supplementary Methods S2–S3 |
| 10b | Statistical analysis | Specify the type of model, all model-building procedures and the method for internal validation | Sections 2.6, 2.7; Supplementary Methods S3–S5 (Firth penalized logistic regression; three pre-specified models; no selection procedure; bootstrap optimism correction) |
| 10d | Statistical analysis | Specify all measures used to assess model performance | Section 2.7 (discrimination, calibration, Brier score, decision-curve analysis) |
| 11 | Risk groups | Provide details on how risk groups were created, if done | Section 3.9 (exploratory triage strategies); Section 3.11 (threshold probabilities) |
| 12 | Development vs. validation | For validation, identify differences from the development data | Not applicable (development only) |
| ***Results*** | | | |
| 13a | Participants | Describe the flow of participants, including the number with and without the outcome | Section 3.1; Figure 1 |
| 13b | Participants | Describe participant characteristics and the number with the outcome | Section 3.1; Table 1 |
| 13c | Participants | For validation, show a comparison with the development data | Not applicable |
| 14a | Model development | Specify the number of participants and outcome events in each analysis | Section 3.10; Supplementary Table S1; Supplementary Methods S4 |
| 14b | Model development | Report the unadjusted association between each candidate predictor and the outcome | Table 1 |
| 15a | Model specification | Present the full prediction model to allow individual predictions | Supplementary Table S1 and the full regression equation given below this checklist |
| 15b | Model specification | Explain how to use the prediction model | Section 3.11; worked examples given below this checklist |
| 16 | Model performance | Report performance measures, with confidence intervals | Section 3.11 (apparent AUC 0.693, 95% CI 0.582–0.802; optimism-corrected AUC 0.645; calibration slope 1.02 apparent, 0.77 corrected; Brier 0.204 apparent, 0.224 corrected against a null value of 0.225) |
| 17 | Model updating | Report the results of any model updating | Not applicable |
| ***Discussion*** | | | |
| 18 | Limitations | Discuss limitations, including non-representative sample and overfitting | Discussion, Strengths and limitations (development in a clinician-selected surveillance-complete subgroup; data-derived age threshold; no clinical outcome data; internal validation only) |
| 19a | Interpretation | For validation, discuss results with reference to performance in the development data | Not applicable |
| 19b | Interpretation | Give an overall interpretation of the results, considering objectives, limitations and other evidence | Discussion; Conclusions (risk-stratification component presented as exploratory and not ready for clinical deployment) |
| 20 | Implications | Discuss the potential clinical use of the model and implications for future research | Discussion, Clinical implications; Conclusions |
| ***Other information*** | | | |
| 21 | Supplementary information | State where supplementary resources can be accessed | Supplementary Methods; Supplementary Tables S1–S2; Data Availability Statement |
| 22 | Funding | Give the source of funding and the role of funders | Funding |

*Items 12, 13c, 17 and 19a apply only to validation or model-updating studies and are not applicable here. Items 10c and 10e of the TRIPOD statement likewise apply to validation studies and are omitted. No external validation was performed; this is stated in the Methods, the Limitations and the Conclusions.*

**Full specification of the prediction model (TRIPOD items 15a and 15b)**

The model was fitted by Firth penalized logistic regression in the surveillance-complete subgroup (105 complete cases, 36 events). Predicted risk is obtained from the linear predictor by the logistic transformation p = 1 / (1 + exp(−LP)), where:

**LP = 2.5808 + 1.0035 × (female sex) − 0.0280 × (preoperative serum sodium, mmol/L) + 1.0389 × (early postoperative hyponatremia) + 0.0198 × (functional adenoma proxy)**

Female sex, early postoperative hyponatremia and the functional-adenoma proxy are coded 1 if present and 0 if absent; preoperative serum sodium is entered as the measured value in mmol/L. Standard errors of the coefficients are 10.4432 (intercept), 0.4342, 0.0750, 0.6095 and 0.4365 respectively; the corresponding odds ratios and confidence intervals are given in Supplementary Table S1. The wide standard error of the intercept reflects the fact that it corresponds to a preoperative sodium of zero and carries no clinical interpretation on its own.

**Worked example 1.** A woman with a preoperative sodium of 138 mmol/L, early postoperative hyponatremia present and a non-functioning adenoma has LP = 2.5808 + 1.0035 − (0.0280 × 138) + 1.0389 = 0.759, giving a predicted risk of 0.68.

**Worked example 2.** A man with a preoperative sodium of 140 mmol/L, no early postoperative hyponatremia and a non-functioning adenoma has LP = 2.5808 − (0.0280 × 140) = −1.340, giving a predicted risk of 0.21.

*These predictions are apparent values from the development sample and are subject to the optimism quantified in Section 3.11 of the main manuscript. Because the optimism-corrected Brier score is essentially equal to that of the null model, the equation should be used to rank patients for targeted POD5–7 surveillance rather than to quote an individual absolute risk, and it requires external validation before clinical use.*
